# Evaluation of Long-lasting insecticidal nets (LLINs) for malaria control in an endemic area in Brazil

**DOI:** 10.1186/s13071-023-05759-4

**Published:** 2023-05-12

**Authors:** Ana Cristina da Silva Ferreira Lima, Allan Kardec Ribeiro Galardo, Josiane Nogueira Müller, Ana Paula Sales de Andrade Corrêa, Kaio Augusto Nabas Ribeiro, Guilherme Abbad Silveira, Andrea Valladão Hijjar, Luiz Guilherme Soares da Roch Bauzer, José Bento Pereira Lima

**Affiliations:** 1 Laboratório de Entomologia Médica, Instituto de Pesquisas Científicas e Tecnológicas do Estado do Amapá (IEPA), Macapá, Amapá Brazil; 2 Laboratório de Fisiologia e Controle de Artrópodes Vetores, Fundação Oswaldo Cruz, Rio de Janeiro, Brazil; 3 Santo Antônio Energia S/A, São Paulo, Brazil; 4 Programa de Pós-Graduação em Medicina Tropical, Instituto Oswaldo Cruz FIOCRUZ, Rio de Janeiro, Brazil; 5 Saneamento Ambiental Projetos e Operações Ltda (SAPO), Rio de Janeiro, Brazil

**Keywords:** Mosquito net, Rondonia, Malaria control, *Anopheles* sp.

## Abstract

**Background:**

Most cases of malaria in Brazil are concentrated in the Amazon region. One of the vector control alternatives recommended by the WHO is the long-lasting insecticidal net (LLIN). This tool is used in the nine federal states of the Brazilian Legal Amazon, where LLINs are essential for reducing vector density and disease transmission as they prevent contact between the mosquito and the individual. The objective of this study was to evaluate the residuality and use of LLIN insecticides in different health regions in a city located in the Brazilian Amazon.

**Methods:**

A total of 17,027 LLINs were installed in the third, fifth and ninth health regions of the municipality of Porto Velho, Rondonia State, Brazil. The LLINs were of two types: Olyset (permethrin), for around the bed, and Interceptor (alphacypermethrin), for around hammocks. The residuality of 172 LLINs was evaluated using cone bioassays to verify the mortality rate of the mosquito *Nyssorhynchus darlingi*, over a period of 2 years. Structured questionnaires on the acceptance and use of LLINs were distributed to the participating population (*n* = 391), covering a total sample of 1147 mosquito nets. The mortality rate was evaluated both in terms of days after LLIN installation and the type of insecticide used. Statistical analyses were based on analysis of variance (ANOVA) and Chi-square and were performed using the SPSS statistical program.

**Results:**

For the *Ny. darlingi* mosquito, Interceptor-type LLINs showed residual efficacy, with mortality rates ≥ 80% during the 2-year study period, as determined by the WHO. In contrast, Olyset-type LLINs were associated with a reduction in mortality rates, with 76% and 45% mortality rates in the last two assessments, which occurred during the last 6 months of the study period. Based on the structured questionnaires, the acceptance rate, i.e. percentage of individuals accepting the permanence of the 1147 LLINs sampled, in the three health regions of Porto Velho was 93.8% (of 1076 LLINs)**.**

**Conclusion:**

The alphacypermethrin-impregnated LLIN was more effective than the LLIN impregnated with permethrin. The results indicate that the correct use of mosquito nets—and consequently the protection of the population—needs to be supported by health promotion actions. These initiatives are considered to be essential for the success of this vector control strategy. New studies that consider the monitoring of the placement of mosquito nets are necessary to provide effective support in the correct use of this methodology.

**Graphical Abstract:**

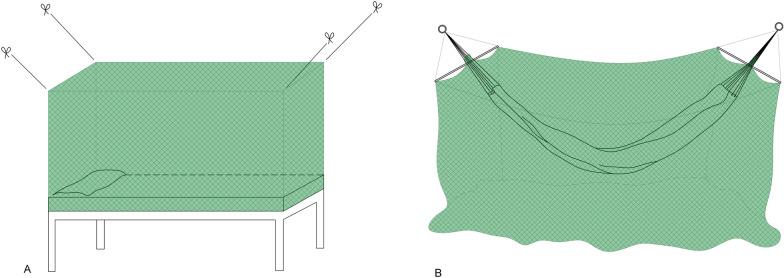

## Background

The use of long-lasting insecticidal nets (LLINs) in malaria endemic areas is a beneficial intervention that reduces the transmission of this disease [1–3]. LLINs are mosquito nets impregnated with insecticidal substances between the polymers that form their fibers. The insecticides used to impregnate the mosquito nets are mainly of the pyrethroid class, with a residual period of 3 years under field conditions, depending on the manner and frequency of washing [4].

Studies on the effectiveness of mosquito nets are more prevalent in the African continent, where malaria is also a serious problem [3, 5, 6]. In one study, LLINs distributed in Malawi were evaluated for the presence and absence of holes after 1 to 2 years; the results showed a greater protection for users who had the LLINs without holes [3]. In other studies, which were carried out in Benin, the results showed that mosquito nets physically protected pregnant women [7] and children who reported sleeping under a mosquito net [8], although the authors emphasize that a poor condition of the LLINs can interfere with their effectiveness. A relevant factor in the African context is the presence of resistance in malaria vectors to the insecticides used in the LLINs [3, 9], making it necessary not only to monitor the bio-efficacy of the nets with resistant mosquitoes [10], but also to update the resistance status in endemic areas to obtain better results from control strategies [11].

In Brazil, malaria cases are concentrated in the Amazon region, where the disease causes considerable social and economic loss to the vulnerable population [12, 13]. The current vector control strategy recommended by the Ministry of Health includes the use of indoor residual spraying (IRS) and LLINs. For mosquito nets, measures are required that include the correct use and monitoring of this technology, as well as the implementation of continued health education actions. A study carried out in the Brazilian Amazon evaluated the use of impregnated mosquito nets 5 years after their distribution and installation. The authors reported that over the long term, a large part of the population did not use mosquito nets properly. It was evident that there was a difference between owning a LLIN and using that LLIN correctly, and that this difference can be minimized through educational measures focused on the benefits of LLINs to intended population [2].

**Fig. 1:**
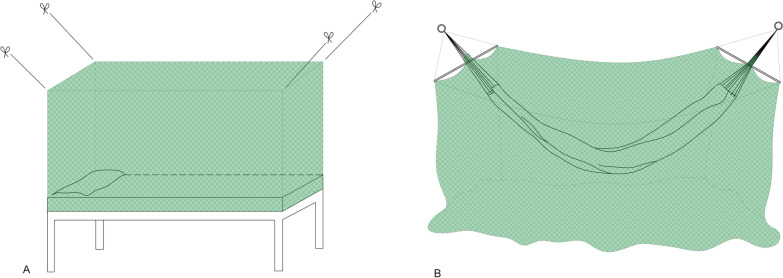
Long-lasting insecticidal net (LLIN) models installed: **a** bed, **b** hammock. Source: drawing by AL Correa

In the northern region of Brazil, part of the population uses hammocks to sleep, as an alternative to beds. This is a legacy from the ancient tradition of indigenous populations in the country [14]. A previous study carried out in Rondonia State (Brazilian Amazon) assessed the impact of LLIN distribution on the annual parasitic incidence (API) [15] of malaria. It is evident that the local reality for making and distributing mosquito nets in the region must be taken into consideration and that different models are essential for covering beds (2 models: double and single) and hammocks (Fig. 1). However, the lack of regional monitoring of vector control measures is a Brazilian reality that needs to be modified based on more studies on malaria in the Amazon region [16, 17].

The aim of the present study was to assess the residuality of insecticides used to impregnate LLINs and describe the patterns of use and care of LLINs based on reports of the users, in three health regions in the municipality of Porto Velho, Rondonia State, Brazilian Western Amazon. This is the first study that specifically evaluates the insecticide residuals of mosquito nets used in hammocks in the field.

## Methods

**Fig. 2:**
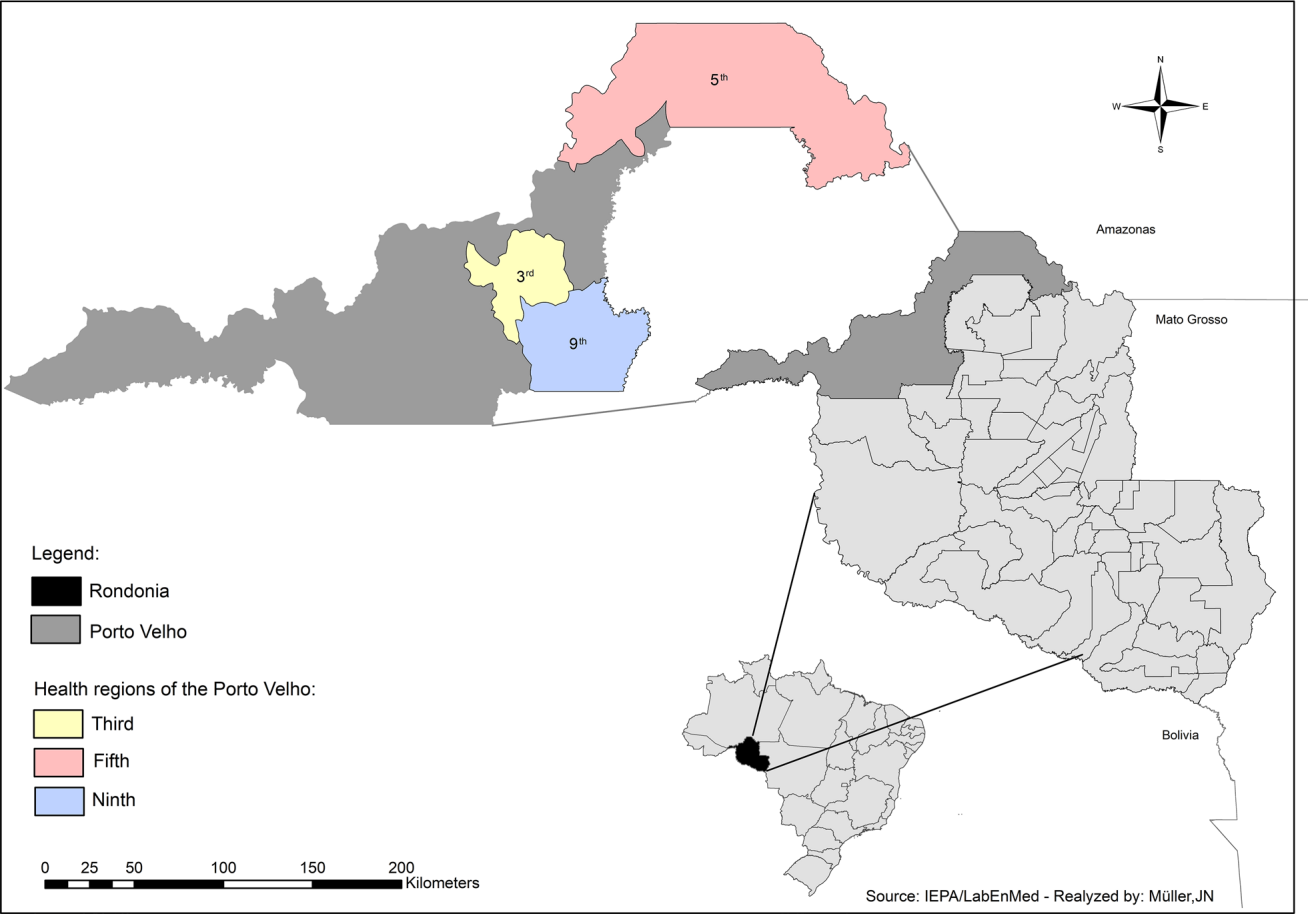
Depiction of the location of the three health regions in Porto Velho, Rondonia State, Brazil, in March 2012. Source: developed by JN Müller

The study was carried out in Porto Velho, the capital city of Rondonia State, which is divided into nine health regions. Three of these health regions, namely the third (Jaci Parana), fifth (Baixo Madeira) and ninth (Rio Pardo) health regions, were selected based on the epidemiological indicators available in the Malaria Epidemiological Surveillance Information System (SIVEP–Malaria) that indicated a high risk of transmission in these regions (Fig. 2).

Health education activities directed at involving the population of the three regions were carried out. This activities included emphasizing the importance of malaria and the correct use of LLINs. Subsequently, 17,027 mosquito nets were installed in homes in March 2012 by Santo Antônio Energia S/A (São Paulo, Brazil) (Table 1). Appropriate instructions for use were provided. Installation was supported by the municipal health department of Porto Velho, within the scope of the public health program of the environmental licensing process (BNDES financing through federal social sub-credit). Two models of LLINs were adopted for the study (Fig. 1). One was a bed model, for either single or double beds); it was rectangular and green (OLYSET®; Sumitomo Chemical Co., Japan), with 2% permethrin insecticide incorporated into the polymers that form the fibers of the fabric (polyethylene). The second model was a green hammock model (INTERCEPTOR®; BASF Chemical Company, Ludwigshafen, Germany), with 0.67% alphacypermethrin insecticide adhered to the fabric's mesh (polyester). Both models have polymers that allow a gradual release of the insecticide from the inside outwards to the surface of the fibers. Residuality assessment was carried out on 172 LLINs (1% of the total sample), 96 of which were of the Olyset® type used for beds and 76 were of the Interceptor® type used in hammocks. The same 172 LLINs were tested at each time point.

**Table 1:** Number of long-lasting insecticidal nets installed in March 2012 in three health regions of the city of Porto Velho, Rondonia State, Brazil

Health region	Residents (*n*)	LLINs installed (*n*)	
		Olyset® (2% permethrin)	Interceptor® (0.67% alphacypermethrin)
Third	15,010	10,932	992
Fifth	1449	707	368
Ninth	4861	3809	219
Total	21,320	15,448	1579

*LLINs* Long-lasting insecticidal nets

Insecticide residuality was assessed on mosquitoes of the species *Nyssorhynchus darlingi* (also known as *Anopheles darlingi*) Root 1926 using biological cone tests on installed mosquito nets [4] [18]. The mosquitos were identified using dichotomous keys [19, 20].

The collection of mosquitoes was carried out at the evaluation sites at the three health regions selected for the study. The capture method established was the protected human attraction technique (PHAT) [21] and was performed the night before the assessment. Mosquitoes were collected using a Castro catcher and were placed in entomological cups. The cups were placed in humid chambers and cotton wool moistened with 10% sucrose solution was provided as food for the females. The tests were carried out according to the availability of mosquitoes at the site, using only those specimens from the evaluated region as a criterion, so that insects do not move between regions and consequently cause interference in bioassays.

The bioassays took place after the installation of LLINs on day 1 and were repeated on the days 90 (3 months), 180 (6 months), 540 (18 months), 630 (21 months) and 720 (24 months) after installation. Random criteria (samples from LLINs were drawn) were used to choose the 172 mosquito nets analyzed, according to the three health regions. It was not possible to perform the 180-day analysis in the fifth health region due to the unavailability of mosquitoes of the species *Ny. darlingi*. Each mosquito net received 12 cones (10 exposed and 2 control group), and five *Ny. darlingi* females were added to each cone. In the control group, a sheet of paper was placed between the cone and the mosquito net, which stopped any contact of mosquitoes with the insecticide. The mosquitoes were exposed for a period of 3 min and later transferred to entomological cups. The insects were packed in a humid chamber and offered a cotton with 10% sucrose solution for feeding. The mosquito mortality reading was performed at 24 h post-exposure. Abbott's correction was performed for bioassays that showed a mortality rate of between 5% and 20% in the control group [22]. The effectiveness of insecticides was evaluated based on: (i) the mortality rate associated with each insecticide as a function of the time elapsed from the installation of the LLINs; and (ii) comparison of the mortality rates among the insecticides used.

### Questionnaire for evaluation of use and care of LLINs

Structured questionnaires were generated from face-to-face interviews performed during home visits. Visits were carried out at 3-month intervals after the installation of mosquito nets for a period of 2 years, totaling seven visits, starting in June 2012. The questionnaire included questions that sought to assess acceptance, coverage, usage and washing patterns of mosquito nets and was adapted from the one used in previous studies [2, 23]. It was distributed to the population in the selected locations. Acceptance was evaluated through the total number of LLINs available in the three health regions 2 years after net installation.

In addition, three criteria were considered to assess the quality of the intervention in the health regions studied: “coverage,” “usage” and “washing.” “Coverage” was evaluated by: (i) LLIN losses, calculated as a difference between the number of LLINs distributed and the number of LLINs found on the day of the interview (no losses, losses ≥ 1); and (ii) number of existing LLINs at home at the time of the interview (1, 2 or > 2). “Usage” criteria were assessed by: (i) LLIN availability period at home (< 12 months, ≥ 12 months); (ii) use frequency within 1 year (use during whole year, only during rainy season, only during drought season); (iii) use frequency within 1 week (do not use at all, 1–3 times/week, 4–7 times/week); (iv) used the night before the interview (yes, no); and (v) how the LLIN was found on the day of the interview (in use, out of use). “Washing” was assessed by: (i) washing every 3 months (yes, no); (ii) use of soap (yes, no); and (iii) let LLIN dry in the shade (yes, no).

### Data analysis

**Fig. 3:**
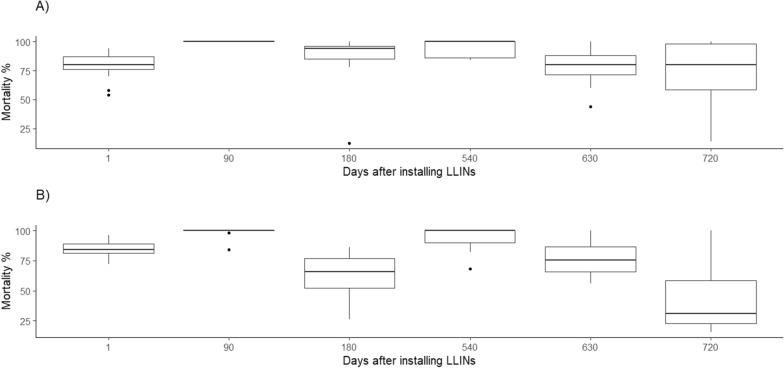
Box-plot of mortality of *Nyssorhynchus darlingi* in contact with LLINs. **a** Interceptor (alfacypermethrin, 0.67%), **b** Olyset (permethrin, 2%). Study period: March 2012–February 2014

Descriptive analyses were based on the number of observations (*n*) and percentages (%). Bivariate analyses were based on Pearson’s Chi-square test for qualitative variables. Quantitative variables were tested based on the analysis of variance test (ANOVA). All analyses were performed using the IBM Statistical Package for Social Sciences (IBM SPSS Statistics for Windows, Armonk, NY, USA); R [22] and RStudio [22] software were used to develop Fig. 3. A *P*-value ≤ 5% was adopted to determine associations with statistical significance.

## Results

The LLINs were installed in an area of the Brazilian Amazon region with a high incidence of malaria. The results demonstrate the effectiveness of mosquito nets over time. Good practices were evaluated by taking into account local reality, and our results showed how the population used mosquito nets during the study period.

Figure 3 shows the variation in the mortality rate and the reduction in the residual effect observed for the two models after 2 years of assessment. A total of 10,366 mosquitoes were used in the bioassays performed at the study participants' homes. At the last evaluation on day 720 (2 years post-installation, the mortality rate of mosquitoes exposed to the Olyset- and Interceptor-impregnated LLINs was 45% and 73%, respectively.

Table 2 shows the ANOVA of the mortality rate observed for each insecticide according to the number of days post-installation of LLINs. For permethrin, mean mortality on day 180 (*P* = 0.007) and on day 720 (*P* < 0.001) was significantly lower than on the day 1. For alphacypermethrin, there was no observed significant difference in mean mosquito mortality rate between day 180/day 720 and day 1.

**Table 2:** Analysis of variance of the average mortality rate observed for each insecticide as a function of the number of days post-installation of long-lasting insecticide nets

Insecticide	Days after LLIN installation	*n*	Mean mortality rate, % (± SD)	*P*
Permethrin	Day 1^a^	15	84.1 (7.1)	
	Day 90	23	99.1 (3.3)	0.061
	Day 180	10	60.8 (21.4)	0.007*
	Day 540	15	93.9 (9.6)	0.554
	Day 630	17	76.1 (12.6)	0.712
	Day 720	16	44.8 (30.5)	< 0.001*
Alfacypermethrin	Day 1^a^	15	79.1 (11.5)	
	Day 90	7	100.0 (0)	0.103
	Day 180	10	89.0 (26.0)	0.981
	Day 540	15	94.7 (7.2)	0.150
	Day 630	15	78.4 (15.0)	1.000
	Day 720	14	73.2 (26.8)	0.944

Study period: March 2012–February 2014

*SD* Standard deviation

*Indicates a significant difference at *P* ≤ 0.05 in mean mortality rate between permethrin- and alfacypermethrin-impregnated LLINs at that time point

^a^Reference

n = number of mosquitoes net evaluated

Table 3 shows the ANOVA of the mortality rate observed at the different post-installation time points as a function of the insecticide used (permethrin vs. alphacypermethrin). Only for day 180 (*P* = 0.043) and day 720 (*P* = 0.012) was the mean mortality rate significantly different between permethrin and alfacypermethrin, being lower for the former insecticide.

**Table 3:** Analysis of variance of the average mortality rate obtained at the various post-installation time points as a function of the insecticide used

Days after LLINs installed (*n*)	Permethrin		Alfacypermethrin		*P*
	*n*	Mean mortality rate, % (± SD)	*n*	Mean mortality rate, % (± SD)	
Day 1	15	84.1 (7.1)	15	79.4 (11.5)	0.158
Day 90	23	99.1 (3.3)	7	100.0 (0)	0.503
Day 180	10	60.8 (21.4)	10	89.0 (26.0)	0.043*
Day 540	15	93.9 (9.6)	15	94.7 (7.2)	0.790
Day 630	17	76.1 (12.6)	15	78.4 (15.0)	0.640
Day 720	16	44.8 (30.5)	14	73.2 (26.8)	0.012*

Study period: March 2012–February 2014

*Indicates a significant difference at *P* ≤ 0.05 in mean mortality rate between permethrin- and alfacypermethrin-impregnated LLINs at that time point

n = number of mosquitoes net evaluated

For the questionnaire, a total of 391 houses, with 1147 LLINs, were selected by convenience sampling, according to the availability of residents in the region. Around 33% of all homes in each health region (3rd, 5th and 9th regions) were selected. In each residence, only one person was invited to answer the questions. Most respondents were female (60.0%), with a low education level: approximately 80% had only elementary schooling (*n* = 309).

The assessments carried out over the 2-year study period showed that most of the population (94.0%) had kept the LLINs in the house where they were installed (*n* = 1076). No LLIN losses were observed for 87.9% of the visited homes; 57.7% participants declared they had slept under the mosquito net between four to seven nights per week, and 52.2% (*n* = 200) reported they had not used the mosquito net the previous night. A total of 40.0% of the sample (*n* = 148) washed the LLINs every 3 months as recommended, while 98.1% declared to have used soap to wash the mosquito net and 68.5% let it to dry in the shade as recommended.

The evaluation of the “coverage,” “usage” and “washing” criteria (Table 4) from the user's point of view showed a lower prevalence of the use of LLINs during dry periods (*P* = 0.047) compared to the periods of rain or over a 1-year period in all regions studied. It was also observed that most users reported not having used LLINs the night before the interview (*P* = 0.013), with the exception of participants in the third health region, where 57% of users reported having used LLIN the night before. Two borderline associations were evident. The first refers to how the LLIN was found during the interview. In most cases, the LLIN was found to be out of use, with the exception of participants in the third region (*P* = 0.078). The second refers to the lower prevalence of washing the LLIN every 3 months as recommended; although not significant, we found a tendency for users to not adhere to this recommendation (*P* = 0.060).

**Table 4:** Association between the washing, use and availability characteristics of long-lasting insecticidal nets and intervention regions

Studied variables	Total LLINs assessed,* N* (%)	Municipal health region			*P*
		Third, *n* (%)	Fifth, *n* (%)	Ninth, *n* (%)	
*Coverage variables*					
LLIN losses					
No losses	343 (87.9)	112 (84.8)	118 (91.5)	113 (87.6)	0.692
Losses ≥ 1	47 (12.1)	20 (15.2)	11 (8.5)	16 (12.4)	
Number of existing LLINs at home					
Up to 1	173 (44.2)	64 (48.5)	53 (40.8)	56 (43.4)	0.527
≥ 2	218 (55.8)	68 (51.5)	77 (59.2)	73 (56.6)	
*Usage variables*					
LLIN availability					
> 12 months	259 (67.4)	82 (64.1)	88 (68.8)	89 (69.5)	0.399
≤ 12 months	125 (32.6)	46 (35.9)	40 (31.3)	39 (30.5)	
Use within 1 year					
Whole year	171 (58.0)	72 (76.6)	50 (47.2)	49 (51.6)	0.027
Rainy season	93 (31.5)	16 (17.0)	40 (37.7)	37 (38.9)	
Drought season	31 (10.5)	6 (6.4)	16 (15.1)	9 (9.5)	
Use frequency within 1 week					
4–7 times/week	207 (57.7)	73 (59.8)	73 (62.9)	61 (50.4)	0.150
1–3 times/week	43 (12.0)	14 (11.5)	10 (8.6)	19 (15.7)	
Do not use at all	109 (30.4)	35 (28.7)	33 (28.4)	41 (33.9)	
LLIN used the night before the interview					
Yes	183 (47.8)	73 (57.0)	59 (45.7)	51 (40.5)	0.013
No	200 (52.2)	55 (43.0)	70 (54.3)	75 (59.5)	
How LLIN was found					
In use	168 (45.7)	64 (53.3)	52 (43.0)	52 (40.9)	0.078
Out of use	200 (54.3)	56 (46.7)	69 (57.0)	75 (59.1)	
*Washing variables*					
Wash every 3 months as recommended					
Yes	148 (40.0)	45 (37.8)	42 (34.1)	61 (47.7)	0.060
No	222 (60.0)	74 (62.2)	81 (65.9)	67 (52.3)	
Soap use as recommended					
Yes	259 (98.1)	80 (96.4)	88 (98.9)	91 (98.9)	0.294
No	5 (1.9)	3 (3.6)	1 (1.1)	1 (1.1)	
Let it dry in the shade as recommended					
Yes	178 (68.5)	60 (74.1)	49 (55.7)	69 (75.8)	0.345
No	82 (31.5)	21 (25.9)	39 (44.3)	22 (24.2)	

Study period: March 2012–February 2014

## Discussion

Long-lasting insecticidal nets are used as a complementary strategy for vector control of *Anopheles* in municipalities of the Brazilian Amazon region where most cases of malaria in Brazil are concentrated [24]. Assessment of the LLINs after their installation in homes is essential, given the extent of the territory covered and specific characteristics of the region. However, few approaches had been described previously in Brazil [2, 15, 25]. Using hammocks for sleeping purposes is one of the characteristics of these local population; consequently any evaluation study of LLINs in the region needs to cover both beds and hammocks to better target the National Malaria Control Program (NMCP). Implications for the capture of *Anopheles* can be a limiting factor in studies of residual time of insecticides in the field, since the colonization of *Ny. darlingi* has only became a reality in Brazil since 2019 [26]. In the present study, rearing mosquitoes in the laboratory was not possible; therefore, it was necessary to use field mosquitoes to carry out the bioassays. In this context, we used mosquitoes from the assessment site that were collected the night before the bioassays and therefore it was necessary to consider the seasonality of the vector in the initial design of the study. However, the low density of mosquitoes in the fifth health region made it impossible to carry out the tests at 180 days after the installation of the LLINs. An evaluation carried out in the Brazilian state of Rondonia after the distribution of LLINs considered the API of the municipalities that received the LLINs and compared it with those not initially included in the malaria control strategy [15]. The study results reported by Vieira et al. showed no difference between municipalities that received LLINs and others that did not receive LLINs [15, 27]. In those studies, Porto Velho, capital city of Rondonia State, was part of the group of municipalities that had not received mosquito nets. However, in 2012, Santo Antônio Energia carried out several local measures, including IRS, thermonebulization (Fog) and installation of LLINs. Thus, we believe that the reduction in the API value observed between the years 2012 and 2013 is related to the control measures of this private company [28], with the monitoring of the LLINs being one of the objectives proposed in the present study. Mosquito nets are installed by public and private initiatives in Brazil, and we therefore propose that the any assessment approaches take this specifically into account.

Our residuality analysis of Olyset-type mosquito nets showed that the mortality rate of *Ny. darlingi* was variable over time. Two years after the installation of the LLINs, the mortality rate was 45% at the last evaluation of the Olyset-type LLINs. This rate differs greatly from the 80% mortality rate considered to be effective by WHO. A longitudinal study to verify the durability of LLINs in Zambia showed, based on cone bioassays (*n* = 80), that after 24 months of evaluation, the average mortality was 51.4% for Olyset-type LLINs [29]. In Tanzania, after a campaign to distribute LLINs, the mortality rate observed in mosquito nets was 55.7% in the evaluation carried out 24 h after installation [30]. In the approaches that compared the mortality rate over the 20 washes proposed by the manufacturer, it was noted that the mosquito net had not reached the recommended mortality [31, 32].

The sustainability of Olyset-type LLINs (2% permethrin) was assessed by Ahogni et al. [33] who evaluated seven different types of LLINs over 12 months and reported that one of the important factors underlying the absence of the mosquito net was donation of the LLINs to people from other homes. In the present study, we observed that the donation of LLINs to other people was also the main reason for the absence of LLINs at the time of the questionnaire. In Mozambique, a lot of damage was identified in the fibers of the mosquito net, with donated mosquito nets being the second-most answered option [34].

In the present study, we observed that Interceptor-type mosquito nets (0.67% alphacypermethrin) presented better results. For this type of LLIN, there was no significant difference in the mean mortality rate when the post-installation days were compared with day 1. In Mozambique [10], mosquito nets impregnated with alphacypermethrin were found to achieve a higher mortality of *Anopheles* than their counterparts impregnated with permethrin (mortality rates of 77.8% and 40.8%, respectively). This result corroborates the results obtained in our study. In regions that have pyrethroid-resistant mosquitoes, Interceptor-type LLINs have been found to have a low mortality rate; this has led to mosquito nets associated with other insecticides being tested in the context of local needs [35]. Monitoring the resistance of malaria vectors in the Americas is important to identify and guide the replacement of insecticides in malaria control.

In terms of the acceptance of mosquito nets, the inhabitants of homes in the present study showed a good adherence to the use of LLINs, with > 90% being at home for monitoring. The authors of a study in Venezuela observed that at 6 months after the distribution of the LLINs, 90% of the study population had accepted using LLINs. These results are similar to those observed in the present study. However, in the long term, adherence may fall, with factors such as the physical integrity of the mosquito net and the educational levels of the population contributing to the rate of use [2, 36].

Our results suggest that there is a level of understanding of the population regarding the importance of mosquito nets as a tool for malaria control. LLINs work by assisting in vector control, functioning as a physical and chemical barrier that prevents not only contact with malaria vector mosquitoes, but also contact with other blood-sucking insects present on the site [37], thereby avoiding the inconvenience of bites and the transmission of other pathogens. Another important aspect of LLINs is the fact that they are protection tools not only for those who are using them, but also for people who do not use mosquito nets and are in close proximity to locations where they were installed. The reason for this is that mosquitoes die when in contact with the insecticides present in the fibers, which implies a reduction in local vector density [38].

Considering the usage criteria, the higher prevalence of individuals who reported having used the mosquito net during the whole year is a factor that reinforces the suitable use of LLINs by the study group. LLINs should be used every night of the week in malaria-endemic regions [2]. More than half of the respondents said they had used mosquito nets up to 7 days per week. However, when approached if they had slept the night before under the LLINs, most of the participants responded negatively.

In this study, 40% of respondents washed the mosquito net every 3 months as recommended, indicating that our group of respondents largely tended to neglect this recommendation. According to the manufacturers of the LLINs and the tests carried out by the WHO Pesticide Evaluation Scheme (WHOPES), the washing of mosquito nets, when established criteria are correctly followed, can assist in the maintenance of mosquito nets, thereby avoiding the loss of residual insecticide and conserving their physical integrity. A study carried out in the Changara District [39], in Mozambique, which assessed the home availability of impregnated mosquito nets and their determinants, showed that the lifespan of mosquito nets can be shorter than expected when washing practices are not carried out properly.

The results of the present study indicate that health promotion actions are essential for the correct use of mosquito nets and, consequently, for the protection of populations from malaria-endemic areas. Educational activities are essential to raise awareness among the beneficiary population and need to be carried out on a permanent basis—not just when mosquito nets are installed. Maximum involvement of the population is important, since previous evaluations carried out in the Amazon region have shown that in the long term there is a reduction in the use of mosquito nets in endemic areas [2, 40]. We conclude that alphacypermethrin-impregnated LLINs were more effective than LLINs impregnated with permethrin in terms of residuality and satisfaction of the beneficiary population. Usage guidelines contributed to the strategy’s success, such as the correct washing frequency, the residuality of the active insecticide in the fibers and the physical integrity of the LLINs. However, future studies that consider the monitoring of the installation of mosquito nets are necessary to effectively assist the correct use of this methodology.

## Data Availability

The datasets used and/or analyzed during the current study are available from the corresponding author on a reasonable request.

## Abbreviations

API: Annual parasite incidence
Fog: Thermonebulization
IEPA: Institute of Scientific and Technological Research of the State of Amapa
IRS: Indoor residual spraying
LLIN: Long-lasting insecticidal net
NMCP: National Malaria Control Program
SAPO: Environmental Sanitation Projects and Operations
SIVEP: Epidemiological Surveillance Information System
WHOPES: WHO Pesticide Evaluation Scheme
